# TiB Nanowhisker Reinforced Titanium Matrix Composite with Improved Hardness for Biomedical Applications

**DOI:** 10.3390/nano10122480

**Published:** 2020-12-10

**Authors:** Joseph A. Otte, Jin Zou, Rushabh Patel, Mingyuan Lu, Matthew S. Dargusch

**Affiliations:** 1 School of Mechanical and Mining Engineering, The University of Queensland, Brisbane 4072, Australia; j.zou@uq.edu.au (J.Z.); r.patel@uq.edu.au (R.P.); m.lu1@uq.edu.au (M.L.); 2 Centre for Microscopy and Microanalysis, The University of Queensland, Brisbane 4072, Australia

**Keywords:** nano-composite, microstructure, nanoindentation, bone implants, powder metallurgy

## Abstract

Titanium and its alloys have been employed in the biomedical industry as implants and show promise for more broad applications because of their excellent mechanical properties and low density. However, high cost, poor wear properties, low hardness and associated side effects caused by leaching of alloy elements in some titanium alloys has been the bottleneck to their wide application. TiB reinforcement has shown promise as both a surface coating for Ti implants and also as a composite reinforcement phase. In this study, a low-cost TiB-reinforced alpha titanium matrix composite (TMC) is developed. The composite microstructure includes ultrahigh aspect ratio TiB nanowhiskers with a length up to 23 μm and aspect ratio of 400 and a low average Ti grain size. TiB nanowhiskers are formed in situ by the reaction between Ti and BN nanopowder. The TMC exhibited hardness of above 10.4 GPa, elastic modulus above 165 GPa and hardness to Young’s modulus ratio of 0.062 representing 304%, 170% and 180% increases in hardness, modulus and hardness to modulus ratio, respectively, when compared to commercially pure titanium. The TiB nanowhisker-reinforced TMC has good biocompatibility and shows excellent mechanical properties for biomedical implant applications.

## 1. Introduction

As our population ages the need for effective bone implants as treatments for arthritis and other joint-related pain is of growing importance [1,2]. Current projections indicate that in the United States replacement surgery for hips and knee arthroplasties will increase by over 600% to more than 4 million operations by 2030 [3]. Many of these surgeries arise due to failure of the original implant and need for a second replacement surgery which can lead to several associated health concerns. Pure titanium (CP-Ti) stands out as a good material for load-bearing joint replacements because of its strong specific properties, corrosion resistance and good biocompatibility [4,5]. However, poor wear resistance of Ti limits its usefulness as the removal of the surface of the implant over time not only contributes to the breakdown of the implant but can also lead to inflammation and related immune responses in the affected area [6]. Attempts to improve strength and wear resistance of Ti through alloying has introduced elements which are prone to leach out over time into the body, limiting their safe use and lifetime [7]. A Ti-based material that retains the aforementioned strengths in biocompatibility and corrosion performance with increased hardness and wear resistance will be a strong candidate as an implant material.

One possible avenue for improving the wear properties is by the surface treatment of Ti, typically with a coating of hydroxyapatite, by methods such as dip coating, plasma spraying and electrochemical deposition [8,9,10,11] amongst others. Hydroxyapatite is a constituent of hard tissues and so interfaces well with the human body when implanted [12]. However, these coatings add an additional cost to the already expensive manufacture of Ti implants and in addition have low bond strength to Ti and are prone to peel off during long-term service [4]. With these facts, producing an uncoated Ti component with strong wear properties is desirable.

Titanium matrix composites (TMCs) have many of the desirable properties for bone implants, with significantly improved hardness and wear properties when compared to CP-Ti. By reinforcing with stiff and strong ceramic particles or fibres, Ti has shown significantly improved strength, hardness and wear resistance [13,14,15,16,17,18,19]. In addition, many of the most promising reinforcements (Ti_x_B, TiC and Ti_x_N) can be manufactured ‘in situ’ meaning that the reinforcement phase is formed during processing (typically facilitated by a reaction at high temperature), which reduces overall process steps and leads to strong bonding at the reinforcement interface [20,21,22]. Among these materials TiB stands out, as it naturally forms a hexagonal whisker morphology which offers an improved strengthening effect when compared to particle reinforcement [23]. TiB is also thermodynamically stable for room temperature operations, leading to stable overall mechanical properties.

There are important concerns about the safety and biocompatibility of whisker reinforced composites for implant materials. Several studies have sought to investigate the cytotoxicity and osteointegration of cells on TiB composites. A 29 vol% TiB whisker reinforced CP-Ti TMC has been tested for biocompatibility and has shown, amongst other results, to have a H index similar to CP-Ti and approximately a third of Ti-6Al-4V indicating that the TiB had no adverse effect on biocompatibility and is not cytotoxic [24]. Similarly, a TiB-TiN Ti6Al4V coating has been shown to have strong biocompatibility and good osteointegration with 15 wt% BN addition [25]. Further, towards dental implant applications, human gingival fibroblasts and osteoblasts on a CP-Ti sample coated with a TiB whisker composite showed similar performance to CP-Ti only indicating their suitability as a biomaterial with up to 10 wt% B addition [26]. Subsequently, studies on the corrosion behaviour of TiB whisker reinforced composites have shown that TiB composites exhibit stronger passive film formation and reduced corrosion when compared to CP-Ti in simulated body fluids (Hank’s solution 37 °C) [27]. This suggests that TiB reinforced composites will remain stable without significant corrosion, preventing leaching of harmful foreign elements. There is very little chance that TiB nanowhiskers will be released into the body. The strong bond between the Ti matrix and TiB produced by an in situ reaction has been shown to significantly reduce fibre pullout and improves the safety of these materials [28]. Further study is required in this area and ‘in vivo’ studies are recommended to identify any potential hazards of TiB nanowhisker-reinforced composites, particularly when subjected to long periods of articulating wear. However, these early studies show that TiB nanowhisker-reinforced TMCs have strong potential to be a safe, viable and long-lasting biomaterial for implant applications.

CP-Ti samples have found strong industrial application as biomedical implants particularly in low wear areas like the femoral stem [28]. However, there are two key problems that need to be addressed. Firstly, a high hardness and wear resistant material is required for high wear and articulating applications such as the femoral head [29]. Secondly, the complexity of coatings coupled with the risk of poor bonding at the surface increases the cost and can reduce the lifetime of coated implants, leading to expensive and dangerous replacement surgery [4]. TiB reinforced matrix composites are in a position to overcome these challenges as a single material can fill the role of a wide range of load bearing implants and with relatively low cost and long lifetime. However, there is a need to develop a thorough understanding of the manufacture of these composites and the optimization of the in situ reaction before they can find useful industrial application in the biomedical industry.

In this study, we demonstrate a cost-effective method to manufacture high aspect ratio TiB nanowhisker reinforced TMCs for load bearing biomedical applications, with mechanical properties investigated by nanoindentation.

## 2. Materials and Methods

In this study, TMC samples were prepared by a process of powder mixing followed by cold compaction and vacuum sintering. CP-Ti gas atomized spherical powders (0–45 μm) were combined with BN hexagonal nanopowders (65–75 nm). One batch of samples was produced with 5 vol% BN and another with 10 vol% BN. Powders were then mixed, compacted and vacuum sintered using the same method as previously reported [15]. Samples were sintered at peak temperatures ranging from 1050 to 1200 °C with the holding times ranging from 2 to 6 h to facilitate the in situ reaction between Ti and BN for the formation of TiB. The furnace heating and cooling rate was fixed at 2 °C/min for all samples. In addition, to reduce the risk of chloride impurities and oxidation, samples were sintered on a bed of yttrium oxide and surrounded with blocks of sponge Ti.

The microstructural characteristics of the manufactured samples were analysed using scanning electron microscopy (SEM, Hitachi SU3500, Brisbane, Australia) and transmission electron microscopy (TEM, Hitachi HF5000, Brisbane, Australia). To prepare samples for SEM imaging, conventional metallographic polishing techniques were used with some SEM specimens etched in a modified Kroll’s solution (86% H_2_O, 10% HNO_3_ and 4% HF) for 30 s. TEM specimens were prepared by two methods. Firstly, bulk samples after sintering were deeply etched with the modified Kroll’s solution for 3 min before being ultrasonicated in ethanol to extract and disperse nanowhiskers in solution. This solution was then dripped onto standard carbon grids, so isolated nanowhiskers could be investigated and measured. Secondly, TEM lamellae were prepared from bulk sintered samples with a FEI Scios Dual Beam FIB using a standard Ga ion beam cut and lift out technique [30]. Sample phases were characterized using a Rigaku SmartLab X-Ray diffractometer (XRD) with a 9 kW rotating Cu anode source. 45 kV and 200 mA were used to generate a 300 µm beam with data recorded by continuous scan from 20–80° 2θ angle in 0.02° increments.

Mechanical properties of the manufactured samples were investigated by nanoindentation tests using a Hysitron Triboindenter with a Berkovich probe. Nanoindentation was chosen in favour of tensile tests because local property measurement facilitates the study of TiB size and microstructural effects, without being affected by other sample variables present in sintered samples, such as porosity. A 12 mN load was chosen as tests conducted with this load in a previous work gave mechanical properties on CP-Ti comparable to that obtained from tensile tests [15]. Oliver–Pharr (OP) analysis was used to determine hardness (H) and reduced elastic modulus (*E_r_*) from the load (P)—depth (h) curve. Elastic modulus (*E*) was then calculated using Equation (1) as shown below, where *v_i_* and *E_i_* are the Poisson’s ratio and elastic modulus of the diamond indenter (0.07 and 1140 GPa) and *v* and *E* are the samples Poisson’s ratio and elastic modulus. Details of OP analysis method can be found in [31]. Samples were tested with at least 100 indents in a 10 × 10 matrix with 10 μm spacing to ensure the result was not affected by the plastic zone of the adjacent indents.

(1) $$\frac{1}{E_{r}}=\frac{1-v^{2}}{E}+\frac{1-v_{i}^{2}}{E_{i}}$$

## 3. Results and Discussion

### 3.1. Powder Mixing

**Figure 1:**
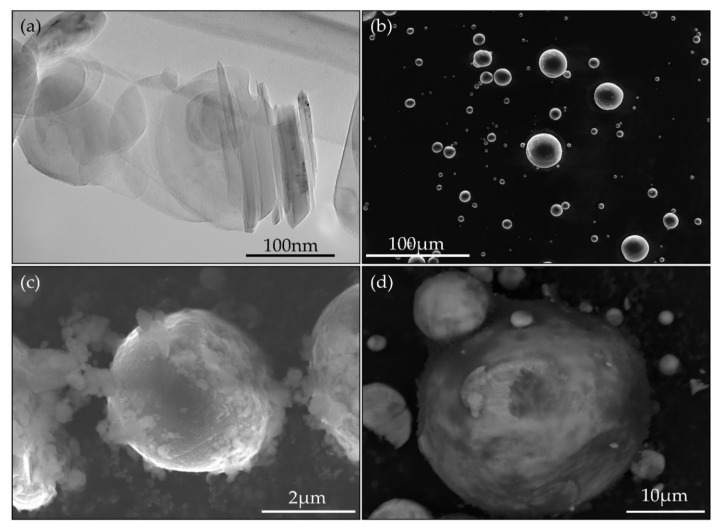
TEM and SEM images of powders before and after mixing: (**a**) TEM image of boron nitride powder as received; (**b**) SEM image of CP-Ti powder as received; (**c**) secondary electron SEM image of powders after mixing; (**d**) back-scatter electron image of powders after mixing.

Powder mixing plays an important role in the manufacture of composite materials [32]. Firstly, proper mixing ensures a uniform distribution of the reinforcement phase in the product part, leading to more uniform mechanical properties. Secondly, when considering in situ processes, well-mixed powders have a higher and more consistent contact area between the two powders which will undergo the in situ reaction. Strong interfacial contact between the reacting particles reduces the risk of any particles remaining unreacted after processing and increases the nucleation rate, acting to refine the microstructure [33]. Figure 1 shows the powders before and after mixing, in which Figure 1a shows the BN nanoparticles as purchased from Nanografi. The BN particles have a plate-like morphology, up to 100 nm in diameter and with a typical thickness of <10 nm. Figure 1b is a low magnification SEM image showing the spherical CP-Ti powders used in this work. Figure 1c,d are secondary electron (SE) and back-scatter electron (BSE) images, respectively, showing the powders after mixing for 4 h in a low energy Turbula shaker mixer with a 3:1 ratio of steel balls [15]. Figure 1c shows the fine BN powders are well distributed across the surface of the CP-Ti and similarly in Figure 1d, where dark patches are indicative of BN. It is clear from this image that BN has been distributed around the surface of CP-Ti particles. Figure 1c also shows some BN plates embedded into the Ti particle which will further promote their in situ reaction. In addition, it is clear that BN nanoparticle agglomerates like those shown in Figure 1a were largely broken up during mixing. Therefore, this mixing process has been effective to distribute BN.

### 3.2. Microstructure

**Figure 2:**
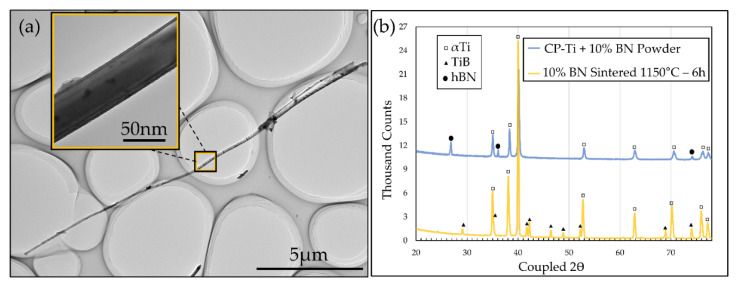
(**a**) TEM image showing a nanowhisker extracted from 10% BN sample after sintering at 1050 °C for 6 h, with inset high magnification image for measuring whisker diameter; (**b**) XRD pattern of 10% BN powder before sintering and after sintering at 1150 °C for 6 h.

Figure 2a is a low magnification TEM image showing a nanowhisker extracted from a sample sintered at 1050 °C for 6 h with an inset high magnification image. This particular nanowhisker had a length and diameter of approximately 18.4 µm and 47 nm, respectively, which was measured along with several others from each set of sintering conditions to obtain the average aspect ratios of the nanowhiskers in each sample. As shown in our previous study these whiskers are TiB [15]. However, to analyse the effect of BN concentration on the chemical nature of the samples, XRD was conducted on a CP-Ti + 10% BN powder sample and then the same powder after sintering at 1150 °C for 6 h with subsequent surface etching (Figure 2b). Each peak in the powder sample was indexed by either hexagonal BN or hexagonal αTi. The XRD peaks in the sintered sample were all indexed by orthogonally-structured TiB and αTi (Figure 2b), leading to two key findings. Firstly, no BN was detected in the samples after sintering, either in XRD or visible in SEM and TEM imaging, showing that all BN has undergone the in situ reaction. Secondly, TiN was also not detected in XRD or SEM and TEM imaging. TiN is regularly present as a product of the reaction between Ti and BN in rapid melting and solidification processes such as laser engineered net shaping (LENS) [25]. However, in slow heating and cooling solid-state processes, such as the furnace sintering employed in this work, no TiN is observed [34,35]. Nitrogen has a wide range of solubility in CP-Ti, >8 wt% at 1050 °C but approaching <0.1 wt% at room temperature [36]. Furnace cooling (2 °C/min) allows the sample to achieve the low temperature equilibrium state with very low solute nitrogen, which was not detected by XRD or EDS in this work or previous studies [15]. The combination of the low solubility of nitrogen at room temperature, the achievement of room temperature equilibrium in slow cooling processes and the absence of detected TiN leads to the conclusion that the nitrogen from BN in this work is lost to the atmosphere during processing.

**Figure 3:**
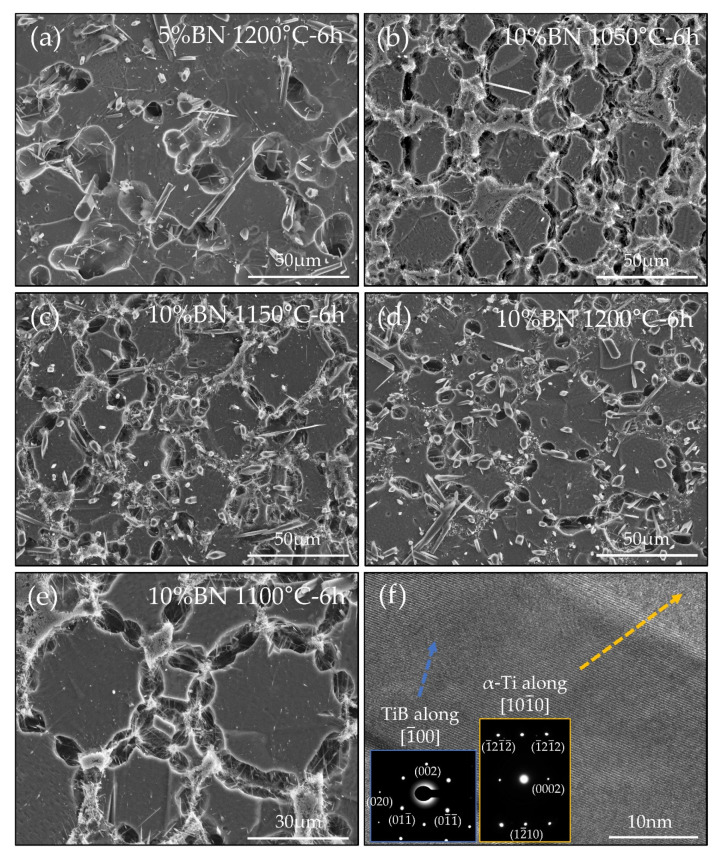
TEM and SEM images showing as-sintered TMC microstructure after etching: (**a**) SEM image of 5 vol% BN TMC sintered at 1200 °C for 6 h; (**b**) SEM image of 10 vol% BN TMC sintered at 1050 °C for 6 h; (**c**) SEM image of 10 vol% BN TMC sintered at 1150 °C for 6 h; (**d**) SEM image of 10 vol% BN TMC sintered at 1200 °C for 6 h; (**e**) SEM image of 10 vol% BN TMC sintered at 1100 °C for 6 h; (**f**) TEM image of FIB cut lamella from 10 vol% BN TMC sintered at 1100 °C for 6 h with inset indexed selected area electron diffraction patterns of TiB and α-Ti.

In order to study the effect of BN concentration SEM images were taken at similar magnification for each of the sintering conditions used. Figure 3a shows a 5 vol% BN TMC sintered at 1200 °C for 6 h and etched for 30 s to reveal details of the microstructure around grain boundaries. For comparison, Figure 3b–d show 10 vol% BN TMCs sintered for 6 h at 1050 °C, 1150 °C and 1200 °C respectively, before 30 s etching. Each microstructure shows Ti grains with nano and microwhiskers of TiB. In order to investigate the effect of reinforcement, two batches of TMC powder were prepared, one with 5 vol% BN and one with 10 vol% BN. The 5 vol% BN sample sintered at 1200 °C includes mostly microwhiskers with average length of approximately 20 µm and diameter of 1 µm (see Figure 3a). Similarly, when the concentration of BN is doubled to 10 vol% with the same sintering conditions, TiB whiskers exhibit a similar structure with average length of approximately 18 µm and diameter of 0.8 µm (see Figure 3d). This comparison shows that up to 10 vol% of BN, the fraction of BN has little effect of the formation and growth of TiB.

In addition to the effect of composition, the impact of processing temperature was investigated. Interestingly, when comparing Figure 3b–d a clear increase in the lateral dimension of TiB whiskers is observable. After sintering at 1050 °C, as in Figure 3b, there are very few TiB whiskers with a diameter greater than 100 nm. Despite significant clustering of TiB, there is a propensity for TiB to remain as nanowhiskers after processing at 1050 °C. TiB in the TMC sample sintered at 1050 °C for 6 h had an average diameter of 50 nm and length of 19 µm. By comparison, as temperature is increased to 1150 °C the number of TiB microwhiskers is increased, while still having significant amounts of TiB nanowhiskers (shown in Figure 3c). The TiB in the sample sintered at 1150 °C for 6 h has an average diameter of 65 nm and length of 23 µm. As temperature is further increased to 1200 °C, as shown in Figure 3d, few TiB nanowhiskers are observed, with the majority coarsened to microwhiskers. It is clear that between 1150 °C and 1200 °C the coarsening of TiB becomes active. This temperature range is consistent with previous studies [14,15], which were conducted on TMCs with lower boron additions indicating that the fraction of TiB present, up to 10 vol%, in the sample has little effect on the critical coarsening temperature.

Figure 3e shows the microstructure of the TMCs produced by this method. After the removal of the Ti in the grain boundary a network structure of TiB interconnecting the Ti grains is observed. The use of low-energy mixing, fine BN and a solid-state sintering operation serves to preserve the structure. As TiB is formed in situ during sintering it grows from the surface of Ti particles into the adjacent particle acting as a grain boundary reinforcement. This microstructure is consistent with that observed in previous works [14,15,37,38,39]. The TiB present in the grain boundary also serves to restrict the grain growth of Ti grains as is commonly observed in slow heating/cooling processes like furnace sintering [37]. The average grain size in each 10 vol% BN sample was approximately 7 µm. A primary detriment to the furnace sintering approach is the coarsening of the grain structure. Therefore, the formation of TMCs by this method, which restricts grain size to a minimum, is of strong benefit.

Figure 3f and insets show a TEM image and selected area electron diffraction (SAED) patterns. Here, a section of a TiB nanowhisker with (011) atomic planes can be observed, indicating that the TiB nanowhisker is a single crystal. The provided SAED patterns provide evidence that the nanowhiskers are TiB with the typical orthorhombic structure, while Ti is present in the HCP structured alpha phase. As expected, no β-Ti was observed in the sample, only α-Ti, due to the slow cooling rate (2 °C/min) allowing for the sample to achieve the equilibrium alpha phase. This thermodynamically stable microstructure is beneficial for long service bone implants as there is less risk of microstructural and therefore property changes over time [40].

### 3.3. Mechanical Properties

α-Ti has excellent corrosion resistance and promotes rapid osteointegration because of a stable TiO_2_ film, where OH^-^ ions present in this surface layer react with ions present in the bone structure like Ca^2+^ and PO_4_^3−^ to aid adhesion [7]. These properties make α-Ti a strong candidate for bone implants. However, the poor fatigue performance and overall low strength have limited the application of CP-Ti and other α-Ti alloys in load bearing implants [7]. A TMC with an α-Ti matrix may offer improved strength and wear properties whilst retaining the corrosion resistance and biological interfacing benefits of α-Ti.

Table 1 presents both results from nanoindentation tests along with mechanical property results from other works. When calculating average E and H, values outside two standard deviations above the mean were rejected. This step was taken as surface damage and contamination can lead to large errors in results when performing nanoindentation [41]. Table 1 shows that our TMC samples exhibit high E and H values when compared to both CP-Ti and other TMCs, even when a higher reinforcement fraction is used. Most notably, the H/E ratio of samples is very high. The ratio of hardness to modulus is directly related to wear resistance and strain to failure [42]. The 10 vol% BN sample sintered at 1150 °C for 6 h has the highest H/E ratio of 0.062, almost twice that of CP-Ti. The ratio H^3^/E^2^, called yield pressure, is associated with yield strength [42,43]. The 10 vol% BN sample sintered at 1150 °C for 6 h has the highest H^3^/E^2^ ratio (0.041 GPa), which is an order of magnitude higher than that of CP-Ti and significantly higher than reported TMCs.

There are several contributing factors to the high performance of our TMCs, one of which is the previously described formation of a network microstructure with reduced grain size. As grain size is reduced, TiB grown in the grain boundary is sufficiently long to grow across the Ti grains, as can be seen in Figure 3. This leads to the improved local property measurement by nanoindentation throughout Ti grains, due to the strengthening effect of TiB. In addition to the increase in hardness and elastic modulus observed, there is an expected increase in bulk material strength as per the Hall–Petch relationship due to reduced grain size [44]. Also, network microstructures have previously been shown to improve both strength and ductility of TMCs [15,21,38,45,46]. Another key factor contributing to the performance of our TMCs is the high aspect ratio of TiB nanowhiskers, particularly in 10 vol% BN samples. The aspect ratio of TiB in samples sintered at 1150 °C and lower is approximately 400. It is well understood that increasing the aspect ratio of the reinforcement phase increases the strengthening efficiency of the reinforcement [23]. However, typically, when the fraction of reinforcement is increased, clustering of the secondary particles prior to the in situ reaction leads to coarsening of the reinforcement phase and, therefore, a decreasing of the strengthening efficiency [33]. In this work, BN fraction showed no effect on the size of TiB, with high aspect ratio TiB nanowhiskers present when 10 vol% BN was used. Figure 3b–d show several fine TiB nanowhiskers grouped together which had not coarsened to microwhiskers. Previous studies have shown that there is a critical temperature for the coarsening of TiB between 1150 and 1200 °C with low TiB fraction (less than 5 vol%) [14,15]. It is clear from this study that TiB retains this critical temperature for coarsening when volume fraction is increased to 10 vol%. Analysis of the formation reaction and coarsening of TiB in detail through in situ microscopy studies may provide necessary information to assist with the design of optimum manufacturing processes, both in solid and melt methods. The combination of increased volume fraction of TiB and maintaining ultrahigh aspect ratio nanowhiskers plays an important role in the enhanced properties of our TMCs.

## 4. Conclusions

To summarize, a TiB nanowhisker-reinforced α-Ti matrix TMC was produced using a method of low energy mixing, cold compaction and furnace sintering. This TMC demonstrated increased strength and wear performance, addressing the two key weaknesses presently facing the use of α-Ti for biomedical implants. Overcoming these challenges is a critical improvement for the development of affordable long-lasting biomedical implants to reduce the number of replacement surgeries required. Analysis showed that TiB is present as nanowhiskers with high aspect ratio in samples sintered at 1150 °C or below with relatively high volume fraction, up to 10 vol% BN. TiB nanowhiskers were observed to coarsen only when the sintering temperature was increased to 1200 °C, even in high volume fractions of BN, shedding light on the in situ reaction process and how it can be manipulated to achieve a refined reinforcement phase. The combination of high aspect ratio TiB in high volume fraction, a fine grain α-Ti matrix and a network microstructure led to an increase in hardness and reduced modulus of more than 300 and 170% respectively when compared to CP-Ti. By improving these key properties while retaining the strong osteointegration and corrosion resistance of an α-Ti matrix these TMCs are a strong candidate for the future of long-lasting load bearing implants. The TMCs produced in this work progress towards addressing the current challenges facing the biomedical implant industry by providing insight into: TiB reinforced TMCs, the in situ reactions at play and their optimized manufacture. Materials such as these will be vital to the next generation of high-performance load-bearing biomaterials.

## Figures and Tables

**Table 1:** Summary of key results from nanoindentation tests, with results from other works for comparison.

Sample Composition	Process Parameters	Hardness/St. Dev (GPa)	Elastic Modulus/St. Dev (GPa)	H/E	H^3^/E^2^ (GPa)	Reference
CP-Ti	1200 °C—6 h	3.43/0.29	94.4/2.3	0.036	0.005	This work
CP-Ti	SLM 120 J/mm^2^	2.39	102	0.023	0.001	[47]
Ti + 5 vol% BN	1200 °C—6 h	7.50/0.70	147.7/15.4	0.051	0.020	This work
Ti + 10 vol% BN	1050 °C—6 h	10.01/1.42	170.7/21.0	0.059	0.034	This work
Ti + 10 vol% BN	1150 °C—6 h	10.48/1.23	167.9/17.2	0.062	0.041	This work
Ti + 5 vol% TiB_2_	SLM 120 J/mm^2^	3.33	122	0.027	0.003	[47]
Ti + 5 vol% TiB_2_	SPS 1150 °C—5 min	4.1	-	-		[48]
Ti + 5 vol% TiB_2_	SPS 1250 °C—5 min	4.3	-	-		
Ti + 25 vol% TiB_2_	SPS 1150 °C—5 min	7.1	-	-		
Ti + 25 vol% TiB_2_	SPS 1250 °C—5 min	10.1	-	-		
Ti + 24 vol%TiB	SPS 1100 °C—5 min	8.2 ^1^	162.6	0.050	0.021	[49]
Ti + 38.5 vol%TiB	1200 °C—5 h	9.7 ^1^	190.5	0.051	0.025	
Ti + 20.6 vol%TiB	HIP 1200 °C, 120 MPa—5 h	8.0 ^1^	169.5	0.047	0.018	

^1^ Originally reported as Vickers hardness, converted to GPa.
